# Macrophage Implication in IPF: Updates on Immune, Epigenetic, and Metabolic Pathways

**DOI:** 10.3390/cells12172193

**Published:** 2023-09-01

**Authors:** Deepak Pokhreal, Bruno Crestani, Doumet Georges Helou

**Affiliations:** 1 Physiopathologie et Epidémiologie des Maladies Respiratoires, Inserm U1152, UFR de Médecine, Université Paris Cité, 75018 Paris, France; 2 FHU APOLLO, Service de Pneumologie A, Hôpital Bichat, Assistance Publique des Hôpitaux de Paris, 75877 Paris, France

**Keywords:** macrophages, pulmonary fibrosis, lung immunity

## Abstract

Idiopathic pulmonary fibrosis (IPF) is a lethal interstitial lung disease of unknown etiology with a poor prognosis. It is a chronic and progressive disease that has a distinct radiological and pathological pattern from common interstitial pneumonia. The use of immunosuppressive medication was shown to be completely ineffective in clinical trials, resulting in years of neglect of the immune component. However, recent developments in fundamental and translational science demonstrate that immune cells play a significant regulatory role in IPF, and macrophages appear to be among the most crucial. These highly plastic cells generate multiple growth factors and mediators that highly affect the initiation and progression of IPF. In this review, we will provide an update on the role of macrophages in IPF through a systemic discussion of various regulatory mechanisms involving immune receptors, cytokines, metabolism, and epigenetics.

## 1. Introduction

### 1.1. Generalities about IPF 

**Figure 1:**
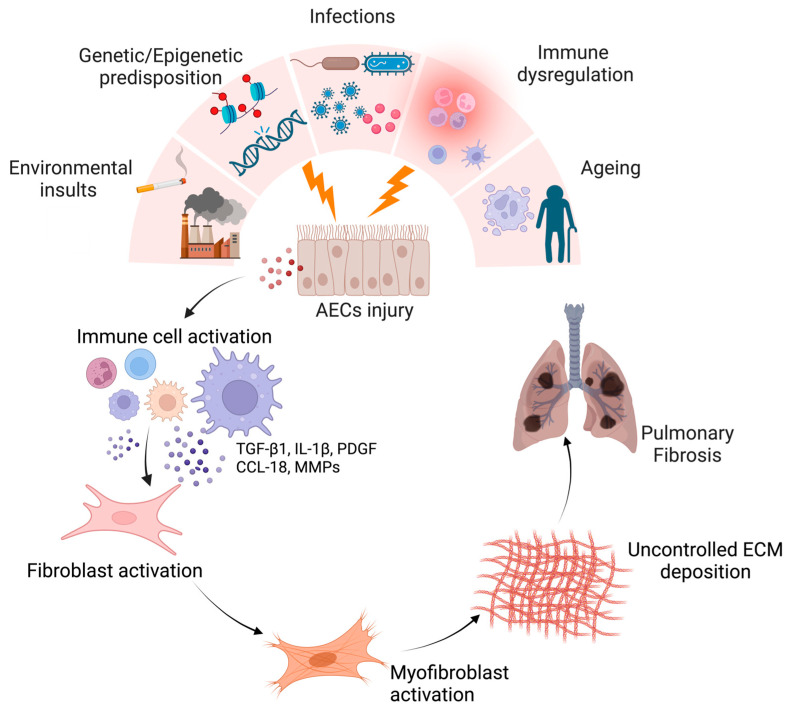
The pathogenic potential of pro-fibrotic macrophages that participate in the onset of pulmonary fibrosis. Repetitive epithelial cell injury caused by a variety of risk factors results in dysregulated epithelial function. AECs release coagulation factors and inflammatory mediators, leading to the activation/recruitment of various immune cells to the site of microinjury. Among these cells, pulmonary macrophages release profibrotic mediators such as TGF-β, IL-1β, PDGF, and CCL18, which lead to the activation of fibroblasts and their differentiation into myofibroblasts to heal the wounded area in the lung interstitium. Defective repair mechanisms and macrophage alternation cause excessive ECM production. Gaseous exchange is significantly decreased due to uncontrolled scarification, which eventually ends up causing respiratory distress. Created with BioRender.com (accessed on 27 August 2023).

IPF is a fatal lung disease characterized by irreversible fibrosis of the lungs, leading to increased cough, dyspnea, and decreased quality of life. It is seen among middle-aged and elderly adults [1,2]. The incidence of IPF ranges from 0.09 to 1.30 per 10,000 persons globally, and the prevalence of the disease ranges from 0.33 to 4.51 per 10,000 persons [1]. Males seem to have higher exposure to fibrotic triggers and are more susceptible to the early development of disease as compared to females [3]. The mechanism behind the pathophysiology of IPF development remains still poorly understood. According to the current paradigm, recurrent alveolar epithelial cell (AEC) injuries occur as a result of predisposing factors such as environmental, genetics, epigenetics, immune, and gerontologic factors, which cause metabolic dysfunction, senescence, aberrant epithelial cell activation, and dysregulated epithelial repair [4,5]. The dysregulated epithelial cells interact with mesenchymal, immune, and endothelial cells via numerous signaling systems, activating fibroblasts and myofibroblasts and causing fibrous content to accumulate in the lungs [5]. Excessive extracellular matrix (ECM) deposition impedes gaseous exchange, eventually resulting in respiratory failure (Figure 1) [4]. Since IPF clinical symptoms overlap with other interstitial lung diseases (ILD), early diagnosis can be challenging and could lead to misdiagnosis. According to guidelines published in 2022 by the American Thoracic Society, European Respiratory Society, Japanese Respiratory Society, and Latin American Thoracic Association (ATS/ERS/JRS/ALAT), the precise diagnosis necessitates observation of clinical characteristics, high-resolution chest imaging, and if necessary lung biopsy, to confirm the pulmonary pattern [6]. The IPF survival was found to be worse than that of many cancers. Its mortality rate is very high, and the median survival of patients is only 3–5 years post-diagnosis [7]. Available treatment options include the use of the anti-fibrotic drugs pirfenidone (PFD) and nintedanib [6]. PFD blocks multiple fibrogenic pathways, most likely through inhibiting transforming growth factor-beta (TGF-β)-mediated fibroblast proliferation and differentiation, whereas nintedanib interferes with several IPF-related pathophysiological pathways and mainly blocks tyrosine kinase receptors to limit the secretion of fibroblast growth factor, platelet-derived growth factor (PDGF), and vascular endothelial growth factor (VEGF) [6,8]. Nevertheless, it has been shown that these medications are only partially successful in treating IPF and have adverse effects such as nausea, anorexia, rash, diarrhea, atherosclerosis, and liver dysfunction [8,9]. Lung transplantation is currently the only curative therapy for IPF [2,6]. However, only a tiny percentage of patients benefit from lung transplants due to the paucity of donors, the difficult surgical procedure, the high cost, and the age of IPF patients. Hence, with the aging population worldwide, IPF is creating a huge socio-economic and healthcare burden in society. Despite significant scientific advancements, the current metrics for diagnosis and medication are still insufficiently sensitive and efficient. Therefore, a better understanding of the molecular mechanisms and various factors that contribute to lung fibrosis is required to deal with this deadly disease.

### 1.2. Main Immune Players in IPF

The immune system’s contribution to the onset of IPF is debatable. For a long time, immune cells were overlooked since the inflammatory hypothesis was not sufficiently supported by prior investigations. Failure of immunotherapies such as interferon-gamma (IFN-ϒ) injections, Tumor necrosis factor alpha (TNFα) neutralization, and immune suppression suggested a limited implication of the immune system in IPF [10]. In the PANTHER-IPF clinical trial, IPF patients were treated with prednisone, azathioprine, and the antioxidant N-acetylcysteine. The combination showed complete failure, leading to an increase in mortality [11,12]. The result interpretation from the latter study supported the notion that the pathogenesis of IPF lacks the immune component. However, a different interpretation can suggest that severe immune suppression in IPF is hazardous, and some immune populations can play important regulatory or anti-fibrotic roles in IPF. Therefore, immunomodulatory strategies that conserve essential immune populations should be adopted instead. It is important to note that translational studies are mostly relying on various animal models to understand the role of different immune cells in pulmonary fibrosis. These models show acute inflammation that progresses to fibrosis, while IPF onset might lack this early inflammatory phase. Additionally, animal models do not reproduce some hallmark pathologic changes seen in IPF, such as predominant lower-lobe fibrosis and clustered cystic changes called “honeycombing” [13]. Despite all this, animal models provide a comprehensive mechanistic understanding of the immune responses that is difficult to obtain in human studies. Interestingly, a common point between numerous studies is that different immune populations can be involved in triggering or alleviating pulmonary fibrosis. The role of different lung immune cells is summarized in Table 1. Because of their dual pro/anti-fibrotic characteristics, macrophages are the most studied cells in this context [14]. Novel technologies, including multiparametric flow cytometry and single-cell RNA sequencing (scRNA-seq), have substantially improved the understanding of lung macrophage heterogeneity. Based on recent human and mouse studies, we will discuss in this review the complex roles of macrophages in IPF from a variety of perspectives, focusing on immune, epigenetic, and metabolic pathways. 

## 2. Macrophages in Lung Fibrosis

Macrophages are mainly antimicrobial phagocytes that build a vital bridge between innate and adaptive immunity. They are found in almost all tissues of the body and play an important role in homeostasis maintenance. The tissue and environment of the organ in which these heterogeneous cells reside determine their functions, such as pulmonary macrophages, adipose tissue macrophages, kupffer cells in the liver, and microglia cells in the central nervous system [14,47]. The respiratory tract and lungs are in direct contact with ambient air, and the alveolar epithelium is, therefore, constantly exposed to particles from both external and host environments. Pulmonary macrophages act as primary immune cells in the lungs and are the first line of defense against inhaled substances. The maintenance of pulmonary macrophage populations is assumed to rely on either the differentiation of blood-derived monocytes or on the proliferation of pre-existing macrophages [22]. Following an injury, damaged epithelial cells contribute to the recruitment of inflammatory cells and fibroblasts in order to promote healing and tissue repair [48]. Pulmonary homeostasis is then reestablished through the activation of apoptotic pathways and phagocytosis by macrophages. However, repeated injury to the alveolar epithelium triggers a dysregulated wound-healing cascade and perturbates macrophage reparative activity. This dysregulation is associated with the release of pro-fibrotic mediators such as TGF-β1, CCL18, Galectin, Connective tissue growth factor (CTGF), and Matrix metalloproteinases (MMPs) that trigger fibroblast migration, proliferation, activation, and differentiation into myofibroblasts [5,38,47]. Pro-fibrotic macrophages have also been reported to transform directly into myofibroblasts through the macrophage-to-myofibroblast transition (MMT) process [26,27,49]. 

### 2.1. Main Macrophage Populations in the Lungs

Two broad sub-populations of macrophages are defined in the lungs and play distinct roles in the fibrotic process: alveolar macrophages (AMs) and interstitial macrophages (IMs).

i-Alveolar macrophages (AMs): AMs derive from embryonic precursors that reside in the lung’s alveoli and are responsible for immune effector properties. Human AM surface markers include CD11b, HLA-DR, CD206, CD169, CD64, and CD141, with minimal CD14 expression [22]. Surface markers found in mice AM include SiglecF, CD11c, CD64, F4/80, and myeloid epithelial reproductive tyrosine kinase (MERTK) [22,50]. AMs are responsible for pathogenic clearance and the release of inflammatory mediators like IL-6, macrophage inflammatory proteins (MIP)-1 and MIP-2, TNF-α, TNF-β, TGF-β, PDGF, etc. [51]. In IPF, circulating monocytes act as a fresh source of macrophages, enhancing the AM pool [52,53]. IPF patients have higher numbers of AMs as compared to healthy individuals and elevated TGF-β secretion, which has been proposed as one of the probable factors causing fibrotic lung disease [26]. As a matter of fact, AMs from IPF fibrotic lungs express more fibrotic genes, such as *IL1RN* (encoding IL-1RA) and *CHI3L1* (encoding chitinase-3-like protein 1), than AMs from healthy lungs in scRNA-seq studies [54]. 

ii-Interstitial macrophages (IMs): IMs are present in the lung’s parenchymal tissue and participate in the maintenance of immune homeostasis in the respiratory system [55]. Human IM surface markers include CD14, CD16, CD169^low^, and CD206 [56]. The identification of IM-specific genes *LGMN*, *MARCKS*, *TMEM37*, and *MERTK* in human IMs using scRNA-seq helps in distinguishing them from other macrophage populations [57]. In mice, depending on the expression of macrophage markers MERTK and CD64, distinct IM subpopulations—IM1, IM2, and IM3 have been identified [21,58]. The ability of IMs to phagocyte particles and to produce reactive oxygen species (ROS) and chemotactic complement is lower than that of AMs, but they express more MHC-II (HLA-DR) to fulfill the role of antigen-presentation [59]. There is limited information on the involvement of IMs in IPF due to their localization in the pulmonary interstitium attached to ECM and the limited access to lung samples.

### 2.2. Macrophage Polarization in IPF

**Figure 2:**
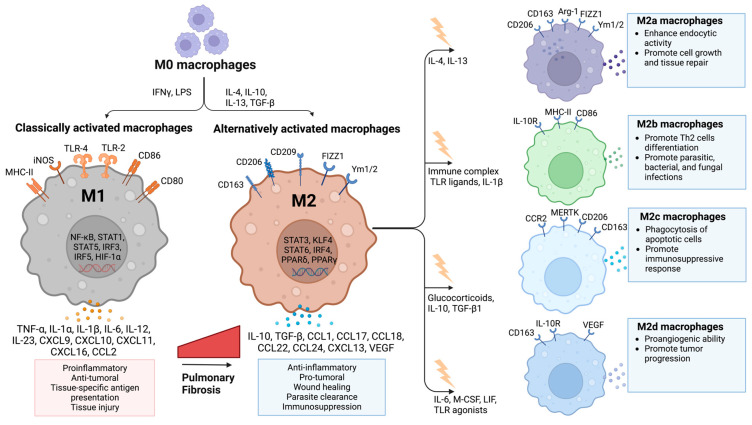
Macrophage polarization. Classically activated macrophages (M1) are generally considered to be pro-inflammatory/anti-fibrotic, and alternatively activated macrophages (M2) are generally anti-inflammatory/pro-fibrotic. M2 macrophages are further classified as M2a, M2b, M2c, and M2d subtypes. Created with BioRender.com (accessed on 27 August 2023).

At the functional level, macrophages are commonly further sub-characterized into two principal phenotypes: classically activated macrophages termed (M1) and alternatively activated macrophages (M2) [23,60]. Although this dichotomy is now the subject of debate in many inflammatory contexts, the M1/M2 functional classification is still helpful in understanding the role of macrophages in IPF. M1 and M2 macrophages are both engaged in inflammatory responses, with M1 macrophages directly associated with pro-inflammatory responses and M2 macrophages predominantly involved in anti-inflammatory responses (Figure 2).

i-Classically activated macrophages (M1): Upon activation with LPS and IFN-γ, naïve M0 or polarized M2 macrophages differentiate into M1 macrophages [23,61]. The expression of CD80, Toll-like receptor (TLR) 4, MHC-II, and CD86 is enhanced in M1 cells [23]. Following stimulation of nitric acid synthase (iNOS), macrophages generate reactive nitric oxide (NO) and produce pro-inflammatory cytokines/chemokines such as IL-1β, IL-12, IL-23, CCL2, CXCL10, and TNF-α [62,63]. The interactions between IFN-γ and its receptors activate Janus Kinase (JAK). Subsequently, JAK phosphorylates the transcription factor signal transducer and activator of transcription (STAT) 1, which then binds to the promoters of class II major histocompatibility complex transactivator (CIITA), iNOS, and IL-12 [27,60]. STAT1 activation is crucial for macrophage polarization to the M1-like phenotype and for protecting against viral infections and intracellular parasites [22]. Additionally, the LPS/TLR4 pathway has been recognized as an important player in the polarization of M1 macrophages. In this pathway, nuclear factor-kappa B (NF-ĸB) and interferon regulatory factor 3 (IRF3) are activated and promote the release of pro-inflammatory cytokines such as IL-6 and TNF-α [23,64,65]. Several investigations have shown that M1 macrophages have anti-fibrotic characteristics. In particular, M1 macrophages generate CXCL10 and MMPs, which promote matrix degradation and inhibit fibrosis [21]. 

ii-Alternatively activated macrophages (M2): M2 macrophages represent an extensively studied population in fibrosis. They arise in response to stimulation with IL-4, IL-10, IL-13, and TGF-β [66]. Human M2 macrophages are characterized by the expression of macrophage mannose receptor (CD206) and CD163, while murine macrophages express arginase-1 (Arg1) chitinase-like proteins Ym-1 and Ym-2 [65]. The polarization of M2 macrophages involves the induction of transcriptional factors and intercellular proteins such as tuberous sclerosis complex 1 (TSC1), stress-responsive activating transcription factor 7 (ATF 7), peroxisome proliferator-activated receptor gamma (PPARγ), ten-eleven translocation (Tet) methylcytosine dioxygenase (Tet2), STIP1 homology and U-Box containing protein 1 (STUB1), Krueppel-like factor 4 (KLF-4) and interferon regulatory factor 4 (IRF4) [14]. They are broadly involved in angiogenesis, tissue remodeling, wound healing, and anti-inflammatory processes [21,60,67]. The M2 phenotype is further classified into 4 subtypes: M2a, M2b, M2c, and M2d. 

The M2a subtype is referred to as wound-healing macrophages that are activated by IL-4, IL-13, fungal, and helminthic infections. M2a cells express Arg1, CD206, CD163, and secrete CCL17, CCL18, and CCL24, which are involved in tissue repair [24,68,69]. M2b cells are known as regulatory macrophages and are induced by IL-1 receptor ligands, immune complexes, and LPS. M2b cells express CD86 and MHC-II and significantly increase IL-1, IL-10, and TGF-β expression upon stimulation [23,57]. M2c cells are known as acquired deactivation of macrophages since they do not achieve M1 polarization [24]. M2c cells are elicited by IL-10, TGF-β1, and glucocorticoids. M2c has high expression of CD206, CCR2, CD163, and MERTK [21]. M2c cells secrete high levels of IL-10 and TGF-β, thus being involved in mediating immunosuppressive responses and tissue remodeling [70]. M2d cells are monocyte-derived macrophages known as tumor-associated macrophages. M2d macrophages are induced by IL-6, M-CSF, leukemia inhibitory factor (LIF), and TLR agonists [23,50]. They highly express VEGF and CD163, secrete IL-6, IL-10, TGF-β, CXCL10, CXCL16, and CCL5, and display a reduced secretion of IL-12. M2d cells are efficient in promoting angiogenesis, matrix remodeling, and adaptive immunity suppression [23,61,71,72]. 

Overall, M2 macrophages constitute the most represented phenotype during IPF progression and might be implicated in triggering lung fibrosis mainly via the production of TGF-β and CCL18 [21,73,74]. Indeed, M2-derived TGF-β promotes lung fibrosis, whereas its depletion ameliorates fibrosis [75]. Recent studies in mice showed that deficiency of the M2-associated small GTPase *Rac2* gene leads to the inhibition of M2 polarization and consequently protects against bleomycin-induced lung fibrosis [76]. In parallel, injecting Rac2-deficient mice with in vitro-polarized M2 macrophages rescued bleomycin sensitivity, while injecting M1 macrophages did not [77]. Consistently, mice overexpressing the transcription factor Fos-related antigen-2 (Fra-2) exhibit increased M2 marker expression on lung macrophages and develop spontaneous lung fibrosis. The deletion of Fra-2, on the other hand, protects mice from bleomycin-induced fibrosis and is linked to reduced M2 marker expression [78]. S100a4, also known as FSP-1 (fibroblast-specific protein-1), belongs to the small Ca^2+^ binding protein family. M2-polarized AMs produce and release S100a4, which enhances lung fibroblast activation and proliferation [79]. Targeting specific macrophage subsets or their polarization in IPF using different strategies is becoming the focus of several investigations. A recent study has demonstrated that intravenous injection of mesenchymal stem cells (MSCs) in bleomycin-treated mice drastically reduces the M2c macrophage population. This suggested that MSC administration can ameliorate pulmonary fibrosis by inhibiting the M2 activation of monocyte-derived macrophages and modulating classical monocytes [25]. The Src homology domain 2 (SH2)-containing tyrosine phosphatase-1 (SHP-1; PTPN6) is a protein tyrosine phosphatase. Using a SHP-1 agonist against lung fibrosis in mice was found to restrict M2 polarization and to prohibit M2-macrophage-orchestrated fibroblast-to-myofibroblast transition [80]. TWIK-related potassium channel (TREK-1, also known as KCNK2) overexpression was also found to be associated with increased M2 phenotype. In parallel, TREK-1 knockdown and pharmacological inhibition restricted the M2 phenotype and diminished bleomycin-induced lung fibrosis [81].

Overall, these findings, along with numerous studies, clearly reveal that M2 macrophages have a crucial implication in the progression and exacerbation of pulmonary fibrosis, while targeting these pro-fibrotic cells and their polarization may help in developing novel therapeutic interventions against IPF.

## 3. Macrophage-Related Mechanisms in IPF

**Figure 3:**
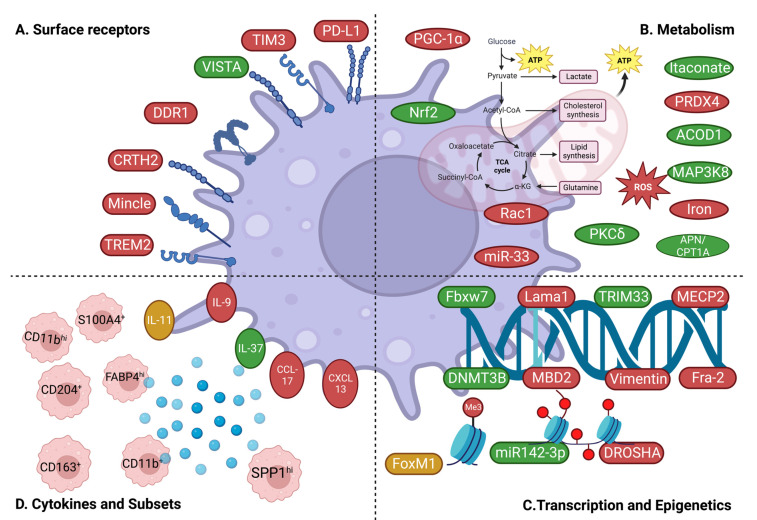
Schematic representation of macrophage-related mechanisms in IPF. Injurious and pro-fibrotic factors are shown in red. Protective /anti-fibrotic factors are shown in green. (**A**) Surface receptor-dependent mechanisms—Above-shown receptors were found to be upregulated in macrophages and implicated in lung fibrosis. (**B**) Metabolism-related mechanisms—Illustrating various fibrosis-related metabolites and metabolic regulators in macrophages. (**C**) Transcriptional and epigenetic mechanisms—Illustrating various transcriptional regulators and epigenetic modifications related to macrophages in lung fibrosis. (**D**) Subsets and macrophage-derived cytokines—Illustrating different cytokines and distinct subsets of lung macrophages in lung fibrosis. Created with BioRender.com (accessed on 27 August 2023).

Although macrophages have long been associated with the progression of pulmonary fibrosis, the mechanisms underlying the responses of these cells in IPF are not fully understood. Due to the heterogeneity and plasticity seen in macrophages from healthy and diseased lungs, it has been difficult to uncover their complex role and interplay with the different pulmonary cells. Recent works have identified novel mechanisms that contribute to the dysregulation of macrophage activity in IPF (Figure 3). This section sheds light on the relevant macrophage-related mechanisms that were described during the last five years.

### 3.1. Surface Receptor-Dependent Mechanisms

Cell surface receptors are membrane-anchored proteins that coordinate macrophage responses to environmental cues and ensure cell-to-cell communication. Alteration of the downstream pathways can dysregulate a range of processes, such as proliferation, migration, phagocytosis, cytokine production, and immune polarization. Therefore, targeting cell-surface receptors is a direct and efficient way to affect cellular signaling and responses. Recently, many studies have demonstrated the capacity of specific receptors to regulate macrophage responses in IPF.

The role of TREM2, a receptor that belongs to the triggering receptors expressed on myeloid cells (TREM), was recently uncovered in IPF. Based on a transcriptomic approach, Luo et al. have demonstrated the upregulation of the *TREM2* gene in BAL cells from IPF patients as compared to healthy donors. Importantly, the expression of TREM2 is predominant in macrophages and positively correlates with the number of M2 cells. In mice, the lack of TREM2 had a protective effect and limited the polarization of macrophages toward an M2 phenotype via the inhibition of STAT6 activity [82]. With a similar approach, Tao et al. have investigated the role of the macrophage-inducible C-type lectin (Mincle) receptor in acute exacerbations of IPF (AE-IPF). Mincle is an innate immune receptor that can recognize diverse lipidic structures derived from pathogens and damaged cells. The expression of Mincle, as well as the proportion of CD14^+^ Mincle^+^ cells, was significantly upregulated in the peripheral blood of AE-IPF and stable IPF patients in comparison with healthy subjects. Moreover, an increased expression of Mincle was detected in the patient’s lung tissues. In the AE IPF mouse model, Mincle deletion attenuated acute inflammation and was associated with a decrease in the percentage of Th17 cells from total CD4 T cells. This decrease was explained by an altered capacity of Mincle deficient macrophages to promote the differentiation of Th17 cells [83].

Cao et al. have recently identified a new regulatory axis for monocyte/macrophage responses in IPF implicating the chemoattractant receptor–homologous molecule expressed on T-helper type 2 cells (CRTH2). The expression of CRTH2 was upregulated on monocyte-derived macrophages in different experimental models, including bleomycin-induced lung fibrosis, TGF-β transgenic overexpression mice, and IL-13 transgenic overexpression mice. The study demonstrated that the interaction between chitinase-3-like 1 (CHI3L1) and CRTH2 promotes pulmonary fibrosis, while CRTH2 deficiency is protective. One of the suggested mechanisms is that CRTH2 promotes a profibrotic phenotype in lung macrophages through CHI3L1–CRTH2 signaling [84]. As collagen deposition represents a key feature in fibrosis, the receptor tyrosine kinase Discoidin Domain Receptor1 (DDR1), which interacts and gets activated by collagens, was studied in IPF. It has been demonstrated that DDR1 inhibition suppresses the progression of bleomycin-induced pulmonary fibrosis and improves the survival rate. DDR1 was mainly activated in the different subsets of lung macrophages. Interestingly, the subsequent mechanistic studies showed that DDR1 promotes Collagen I stimulation-triggered inflammasome synthesis and activation in macrophages. This implicates an enhanced M1 polarization. DDR1 activation in macrophages was also confirmed in the lung tissues of IPF patients as compared to healthy donors, strengthening, therefore, the translational aspects of these findings [85].

Previously associated with T cell inhibition, the expression of several immune checkpoints was reported on macrophages. Among these receptors, the V-domain immunoglobulin suppressor of T-cell activation (VISTA) is expressed on macrophages and reduces proinflammatory cytokines production. In parallel, VISTA agonist enhances the secretion of anti-inflammatory mediators, including IL-10 [86]. Interestingly, a recent study by Kim et al. has characterized the expression of VISTA on macrophages in IPF and investigated its role in the context of pulmonary fibrosis. Using publicly available scRNA-seq data, they have revealed the upregulation of the VISTA coding gene (*VSIR*) in monocyte-derived AMs from IPF patients as compared to healthy donors. VISTA is also inducible on AMs in bleomycin-induced lung fibrosis. The experimental approaches in VISTA knockout mice suggested a protective role for VISTA in the bleomycin model of pulmonary fibrosis. Importantly, the use of VISTA agonists reduced collagen deposition as well as the levels of relevant innate immunity mediators [87]. Taken together, this work highlights the involvement of VISTA in pulmonary fibrosis, while further studies are required to uncover the underlying mechanisms and to understand the role of VISTA in IPF. The implication of another immune checkpoint, T-cell immunoglobulin domain, and mucin domain-3 (TIM-3) was also investigated in IPF. Wang et al. revealed that the levels of TIM-3 are increased in IPF patients peripheral blood mononuclear cells. In mice, the overexpression of TIM-3 was associated with the exacerbation of pulmonary fibrosis. Unexpectedly, TIM-3 is mainly expressed on AMs in fibrotic lungs. Consistently, in vitro assays showed that TIM-3 enhances the production of TGF-β1 and IL-10 by macrophages. Depletion and adoptive transfer experiments using TIM-3 transgenic mice confirmed that TIM-3 induction on AMs potentiates pulmonary fibrosis [88]. At the level of immune checkpoint ligands, Jovanovic et al. have described the expression of PD-1 ligand (PD-L1) in IPF. Based on histological analyses, they showed that PD-L1 is overexpressed on AMs in IPF patients when compared to healthy donors, in correlation with increased levels of soluble PD-L1 in the serum [89]. Overall, the available literature on immune checkpoint receptors in IPF is controversial and remains insufficient to suggest these receptors as promising targets. The PD-1/PD-L1 axis, which promotes immune exhaustion in cancer, has been studied in IPF, but its role remains conflicting. Additionally, a closer highlight on TIM-3 and VISTA roles in IPF indicates that inhibitory checkpoints might play opposite roles in IPF and, therefore, targeting these receptors requires different strategies between inhibition and agonistic activation. While there is an overlap in pathogenic mechanisms between IPF and cancer, further longitudinal studies are warranted to provide a clear picture of the possibility of targeting these receptors in fibrosis.

### 3.2. Metabolism-Related Mechanisms

Metabolic pathways are used by immune cells to provide optimal energy stores to ensure their survival and fulfill their effector functions. In macrophages, as in most immune populations, cell activation and cytokine production are closely dependent on the mechanisms of energy production. Particularly, the metabolism of macrophages can strongly affect their polarization, and metabolic adaptations are crucial to shift macrophage responses in specific inflammatory contexts. In IPF, changes in fatty acid composition perturbate the homeostasis of alveolar type II epithelial cells, while it is believed that macrophages and fibroblasts rely on glycolysis to meet their metabolic requirements [90]. When glycolytic pathways are activated, lactate accumulation occurs in the pulmonary microenvironment and enhances fibroblast proliferation, differentiation, and collagen synthesis, aggravating fibrotic remodeling [91,92]. Tightly linked to metabolic reprogramming, mitochondria dysfunction, and oxidative stress are also prominent features of profibrotic responses. Iron-dependent mitochondrial dysfunction is one of the main mechanisms that are highlighted for their contribution to the pathogenesis of pulmonary fibrosis. Recent works have highlighted several metabolic events involved in macrophage polarization in IPF, providing, therefore, a new promising angle based on immunometabolism. 

The family of microRNA-33 (miR-33) is known to control cellular lipid metabolism in macrophages and to repress genes involved in cholesterol efflux and fatty acid oxidation. A recent study by Ahangari et al. revealed the role of the miR-33 family in IPF through the reprogramming of macrophage metabolism. The study first showed increased levels of miR-33 in monocytes/macrophages isolated from the patient’s BAL and lungs. Using a myeloid-specific miR-33–knockout mouse, it has been demonstrated that miR-33 enhances the profibrotic contribution of myeloid cells in bleomycin-induced lung fibrosis. Interestingly, the loss of miR-33 leads to an increased mitochondrial function in AMs. This is associated with the induction of autophagy and mitophagy to limit the metabolic alterations in the context of bleomycin injury. Last, pharmacological inhibition of miR-33 in mouse and human ex vivo models suggested a new potent therapeutic strategy against pulmonary fibrosis based on a metabolic reprogramming of macrophages [93]. Wu et al. have investigated the effect of adiponectin/carnitine palmityl transferase 1A—(APN/CPT1A-) mediated fatty acid metabolism on lung fibrosis progression. They demonstrated in vitro that activated macrophages can suppress fatty acid oxidation in fibroblasts and decrease autophagy. In contrast, the activation of APN/CPT1A signaling enhances fatty acid metabolism and reverts the effect of M1 macrophages on fibroblasts. In particular, APN/CPT1A activation decreases the expression of TGF-β, α-SMA, and Collagen I in fibroblasts and ameliorates autophagy. Consistently, the induction of APN/CPT1A signaling limits pulmonary fibrosis in a rat model of pulmonary fibrosis. Taken together, this study suggests that macrophages could potentiate the fibrotic activity of fibroblasts via the alteration of their fatty acid metabolism in IPF [94]. Among other functions, protein kinase C delta type (PKCδ) is considered a metabolic regulator for insulin sensitivity, glycolysis, and mitochondrial respiration. Wang et al. studied the implication of PKCδ in the pathogenesis of pulmonary fibrosis. They demonstrated that IPF patients exhibit higher phosphorylation of PKCδ in the lungs as compared to healthy controls. In mice, PKCδ deficiency worsens bleomycin-induced pulmonary fibrosis and potentiates lung inflammation. In vitro assays showed that the lack of PKCδ in macrophages increases the secretion of IL-1β, IL-6, TNF-α, and IL-33. PKCδ does not have any significant effect on the phosphorylation of JNK, ERK, and p38 MAPK; however, it suppresses the NF-κB pathway along with direct phosphorylation of A20 [95]. Although this study did not investigate the effect of PKCδ deficiency on macrophage metabolism in IPF, it clearly highlighted this metabolic regulator as a potential target against IPF.

The mevalonate pathway is an essential metabolic pathway that provides the cells with sterol and non-sterol isoprenoids. Larson-Casey et al. reported in IPF a link between the flux of the mevalonate pathway and the posttranslational modification of the Rho GTPase Rac1 in macrophages. They observed that the activity of mitochondrial Rac1 is upregulated in BAL cells from IPF patients in comparison with healthy donors. The increased activity of Rac1 in mice fibrotic lungs and human BAL was associated with an increase in Arginase 1 activity. Interestingly, the geranylgeranylation of Rac1 is required to enhance Rac1 activity and induce a profibrotic phenotype in macrophages. This polarization depends on mitochondrial ROS. While statins promoted pulmonary fibrosis via Rac1 activation in macrophages, deletion of Rac1 has a protective effect. Taken together, this study indicated that the posttranslational modification of Rac1 through the mevalonate pathway leads to profibrotic polarization of macrophages in IPF and suggested, therefore, a promising metabolic target [96]. In another study, Larson-Casey et al. uncovered a novel mechanism related to oxidative metabolism. This study revealed that BAL cells from IPF patients display upregulated mitochondrial biogenesis when compared to healthy donors. This necessitates an increased activity of the positive regulator PPARɣ coactivator (PGC)-1α to enhance mitochondrial electron transport. Importantly, Akt1-mediated mitochondrial ROS potentiates PGC-1α activity in monocyte-derived macrophages and provides these cells resistance to apoptosis. In parallel, conditional deletion of PGC-1α in monocyte-derived macrophages dampens bleomycin-induced fibrosis in mice [97]. This indicates that the inhibition of the metabolic regulator PGC-1α in macrophages could be a promising strategy against IPF. 

Itaconate is an inflammation-related mitochondrial metabolite that steers oxidative stress in macrophages through metabolic reprogramming. A study by Ogger et al. elucidated the effect of Itaconate on macrophage fibrotic response. They revealed a decrease in itaconate and itaconate-synthesizing cis-aconitate decarboxylase (ACOD1) levels in AMs from IPF patients in comparison with controls, suggesting a dysregulated axis. The lack of ACOD1 leads to the exacerbation of lung fibrosis in mice. In particular, ACOD1-deficient AMs promote the profibrotic response. Importantly, itaconate modulates the metabolism of macrophages via the upregulation of oxidative phosphorylation, as revealed by the seahorse mitostress test. Consistent with the observed anti-fibrotic effects, mice treatment with exogenous itaconate limits collagen deposition and improves lung functions in bleomycin-induced pulmonary fibrosis [98]. Cyclooxygenase-2 (COX-2) regulates the metabolism of innate immunity mainly via the production of prostaglandins (PGs). Furthermore, PGs can affect macrophage polarization through the increase in mitochondrial oxidative phosphorylation. Zannikou et al. identified the MAPK kinase MAP3K8 as a regulator of PGE2 in the context of lung fibrosis. They observed that MAP3K8 expression is altered in fibrotic lungs from IPF patients and bleomycin-treated mice. Importantly, MAP3K8 deficiency in macrophages is associated with enhanced recruitment of inflammatory cells to the lungs and, consequently, worsened pulmonary fibrosis in mice. Moreover, MAP3K8 deficiency disturbs cellular metabolism in bleomycin-induced pulmonary fibrosis, as revealed by the reduced expression of COX-2. Consistently, PGE2 levels are decreased in the absence of MAP3K8, promoting, therefore, the exacerbation of pulmonary fibrosis. Taken together, an altered MAP3K8 signaling worsens pulmonary fibrosis via a COX-2-dependent metabolic mechanism, highlighting a potential protective role for MAP3K8 in IPF [99]. In the same context, Tsitoura et al. elucidated mitochondrial homeostasis in AMs. Results from this study demonstrated an increased level of mitochondrial ROS in AMs associated with an altered transcriptional phenotype and an increase in the expression of the scavenger receptor CD163. Moreover, dysmorphic and contained disorganized cristae were detected in AMs from IPF patients using transmission electron microscopy. The potential effect of mitochondrial activity impairment on IPF progression highlights the importance of a new class of targets in limiting the profibrotic response of macrophages [100].

Given the critical contribution of ROS in lung fibrosis, several studies investigated new oxidative pathways in macrophages. Hanaka et al. compared the seral levels of peroxiredoxin 4 (PRDX4), an antioxidant enzyme, between stable and exacerbated IPF patients. Interestingly, the progression from the stable to the acute form is associated with a significant increase in the levels of PRDX4 in correlation with the increase in relevant biomarkers, including Krebs von den Lungen-6 and surfactant protein D. In mice, the overexpression of human PRDX4 worsens the progression of lung fibrosis and reduces the survival time following bleomycin injury. Histological analysis confirmed that PRDX4 is mainly expressed in AMs and epithelial cells, suggesting a new diagnosis and therapeutic target in IPF related to macrophages [101]. On the opposite side, a study by Liu et al. elucidated the therapeutic effect of NF-E2-related factor-2 (Nrf2) modulation in macrophages. Nrf2 is a major regulator of cellular oxidative and electrophilic stress that enhances the production of antioxidant enzymes such as superoxide dismutase (SOD) [102]. The study showed that the accumulation of M2 macrophages in bleomycin-induced fibrosis is associated with a decrease in the levels of SOD and excessive production of malondialdehyde (MDA), a principal pro-oxidant molecule formed during the peroxidation of polyunsaturated lipids. ROS-responsive liposomes potentiating Nrf2 activity in macrophages via the delivery of dimethyl fumarate were designed and tested in the context of lung fibrosis. First, these liposomes were able to inhibit fibroblast-to-myofibroblast differentiation in vitro through the activation of the Nrf2 axis in macrophages and therefore limited the production of collagen. In vivo, the activation of Nrf2 limited the accumulation of macrophages and dampened lung fibrosis [103]. Taken together, these studies confirm the capacity of macrophages to regulate the progression of lung fibrosis via their oxidative systems.

Iron is a key micronutrient needed for the metabolic and bioenergetic functioning of cells. Excessive iron accumulation in IPF may induce iron-driven oxidant injury in epithelial cells, leading to mitochondrial dysfunction and worsening lung functions [104,105]. The link between iron accumulation and macrophage polarization toward a profibrotic activity in IPF has gained interest during the last decade. Lee et al. revealed that the intracellular level of iron in AMs, as well as iron-dependent ROS, are upregulated in IPF patients in comparison with healthy donors. Consistently, iron chelation reduces the accumulation of ROS in murine AMs, while the induction of iron-dependent ROS enhances their pro-inflammatory phenotype [106]. In line with the abovementioned study, Allden et al. elucidated the mechanisms related to transferrin receptor 1 (CD71) expression by AMs in IPF. In normal conditions, transferrin can bind circulating iron to limit iron-mediated oxidative stress. Of interest, this study showed that the proportion of CD71^−^ AMs increases in IPF patients as compared to healthy donors. Moreover, CD71^+^ and CD71^−^ AMs were phenotypically and functionally different. In particular, CD71^−^ cells have a pronounced profibrotic phenotype with an upregulated expression of *IL10, CCL3*, and *VEGFA* [52]. This indicates that CD71^+^ cells play a protective role in IPF via the sequestration of free iron and suggests targeting the CD71^−^ alveolar macrophage subset in IPF. Ali et al. expanded upon clinical data and demonstrated that iron accumulation in transferrin receptor 2 (Tfr2) mutant mice and homeostatic iron regulator deficient mice promotes collagen deposition and deteriorates lung functions. Furthermore, iron levels were increased in bleomycin-induced pulmonary fibrosis, while a panel of iron-related genes was affected. Importantly, the highest amount of iron was detected in macrophages, and the number of CD71^+^ macrophages increased significantly. This suggested that the accumulation of CD71^+^ macrophages is linked to higher cellular sequestration of iron. Further analysis showed that CD71^+^ cells have a dominant M2-like phenotype characterized by an enhanced expression of M2 genes such as *Il10*, *Arg1,* and *Timp1*. Iron-chelating molecules were efficient in reducing the number of CD71^+^ macrophages and abrogating pulmonary fibrosis [105].

Altogether, the key link between macrophage metabolic dysregulation and fibrosis development is getting more recognized in the IPF field. Understanding the different aspects of macrophage metabolism in IPF might, therefore, open the door to potential therapeutic strategies that delay the progression of fibrosis via metabolic reprogramming. 

### 3.3. Transcriptional and Epigenetic Mechanisms

Several factors regulate gene activity and whether genes are turned on or off, including transcription factors and epigenetic modifications. Transcription factors are proteins that recognize specific DNA sequences to control the initiation of gene transcription. Epigenetic marks, such as DNA methylation and histone modifications, could modulate regions of the genome to promote gene activation or repression [107,108]. Of note, the epigenetic machinery could block the binding of transcription factors to gene promoters. Numerous studies have emphasized that histone changes, including acetylation, methylation, and ubiquitination, affect the expression of genes responsible for fibrotic processes. Targeting DNA methylation and histone-modifying enzymes has shown promise in preclinical models, indicating the potential for epigenetic treatments to slow down the progression of IPF. These therapies have the potential to halt or even reverse fibrotic remodeling by restoring the balance of gene expression in macrophages. For instance, new molecules that could modulate epigenetic modifications, such as FibroGen-FG-3019, are being tested in clinical trials. In this section, we will highlight recently described transcriptional and epigenetic mechanisms that steer macrophage activity in IPF.

DNA methyltransferase (DNMT)3B is an oxygen-sensitive enzyme that ensures de novo DNA methylation via its catalytic role and, therefore, participates in the maintenance of chromosomal homeostasis. A recent study by Qin et al. investigated the impact of DNA methylation on macrophages focusing on DNMT3B. They first showed that DNMT3B deficiency enhances macrophage polarization toward the M2 phenotype in response to IL-4 and TGF-β1. The inhibition of *Arg1* promoter methylation is one of the suggested mechanisms that could explain the effect of DNMT3B on macrophage polarization. Then, using a myeloid cell-specific DNMT3B deficient mice, they demonstrated in the bleomycin-induced pulmonary fibrosis model that the lack of DNMT3B increased the recruitment of fibrotic AMs (SiglecFlowCD11bhi) as compared to classic alveolar macrophage population (SiglecFhiCD11blow). This was associated with an increased expression of M2 genes such as *Arg1*, *Fizz1*, *Pdgfa,* and *Mmp8*. Consistently, DNMT3B deficiency in myeloid cells exacerbates pulmonary fibrosis, suggesting a protective role for DNMT3B in IPF via the regulation of macrophage polarization [109]. 

The methyl–CpG-binding domain (MBD) proteins belong to the epigenetic machinery. Indeed, they bind to the methylated CpG DNA and promote the formation of a suppressive complex. Wang et al. studied the effect of MBD2 on macrophage activity in IPF. Interestingly, MBD2 is highly expressed in CD206^+^ macrophage lungs from IPF patients as compared to an undetectable expression in healthy donors. Using myeloid cell-specific MBD2 deficient mice, it was demonstrated that MBD2 deficiency decreases collagen deposition and significantly ameliorates bleomycin-induced fibrosis. Further analyses showed that the lack of MBD2 contributes to the attenuation of TGF-β1 secretion by lung macrophages as well as the downstream pathways. Other results supported the notion that MBD2 deficiency dampens the M2 polarization via the inhibition of the PI3K/Akt signaling pathway. Consistently, a therapeutic strategy based on the administration of *Mbd2* siRNA liposomes exhibits a protective effect against bleomycin-induced pulmonary fibrosis [110]. Within the family of epigenetic regulators, the methyl-CpG-binding protein 2 (MECP2) can also bind CpG islands at the methylation sites. Mou et al. have revealed that the expression of MECP2 is specific to macrophages in fibrotic lungs from IPF patients, while the expression is undetectable in healthy subjects. Similar results were obtained in the bleomycin-induced lung fibrosis model, confirming the induction of MECP2 in fibrotic lungs. In vitro results indicated that MECP2 enhances the M2 phenotype via the induction of IRF4 in macrophages. Interestingly, *Mecp2* siRNA-loaded liposomes targeting lung macrophages are able to suppress pulmonary fibrosis in mice, suggesting a novel epigenetic target in IPF [111].

The citrullination of several structural proteins, including vimentin, could represent a key mechanism for the epigenetic control of the innate immune system under pathological conditions [107,112]. Li et al. have addressed the involvement of vimentin citrullination in IPF development. They first measured in IPF patients elevated levels of two cigarette smoke molecules, cadmium (CD) and carbon black (CB). Importantly, the levels of these pollutants were correlated with the amount of secreted citrullinated vimentin by lung macrophages and with the severity of the disease. Three-dimensional lung pulmospheres showed the capacity of citrullinated vimentin to induce an invasive subtype of fibroblasts. Furthermore, citrullinated vimentin promotes profibrotic cytokine production via the activation of the TLR4/NF-κB signaling pathway [113]. Altogether, this study highlighted macrophage-derived citrullinated vimentin in IPF as a critical danger signal for the activation of fibroblasts.

The importance of DNA methylome-encoded information in AMs during lung fibrosis was assessed by McErlean et al. DNA methylation profiling uncovered the epigenetic heterogeneity of AMs in IPF. In particular, data suggest that aberrant AM metabolism during IPF may be partly related to discrete modifications in macrophage methylome. Indeed, differentially regulated regions were identified in metabolism-related genes, such as *LPCAT1* (lysophosphatidylcholine acyltransferase 1) and *PFKFB3* (6-phosphofructo-2-kinase/fructose-2,6-biphosphatase 3). Importantly, the described epigenetic modifications correlate with the disease severity and confirm the capacity of the epigenetic machinery to orchestrate different aspects of macrophage responses [114]. 

Tripartite motif-containing 33 (TRIM33) is an E3 ubiquitin ligase known as a transcriptional repressor. Given the negative effect of TRIM33 on TGF-β/SMAD signaling, Boutanquoi et al. investigated its role in IPF. The expression of TRIM33 is mainly upregulated in myofibroblasts and macrophages in IPF patients in comparison with control subjects. Similar results were obtained in rats and mice after bleomycin injection. Using bone marrow-derived macrophages, it has been shown that TRIM33 deficiency potentiates the production of TGF-β1 as well as other fibrotic mediators in response to bleomycin stimulation. Consistently, hematopoietic-specific TRIM33 deficient mice exhibited an aggravated fibrotic progression, confirming the protective role of TRIM33 in pulmonary fibrosis, at least partly through the regulation of the TGF-β1 pathway in macrophages [115]. A study conducted by He et al. has recently unpinned the role of another E3 ubiquitin ligase F-box and WD repeat domain–containing 7 (Fbxw7) in IPF. The mRNA expression of *Fbxw7* is downregulated in the BAL cells of IPF patients in comparison with healthy donors. In mice, the expression of Fbw7 is lower in circulating monocytes and lung macrophages after 21 days of bleomycin administration. To reveal the role of Fbxw7 in lung macrophages, a myeloid-specific deficient mouse was created. This mouse displayed an excessive deposition of collagen in response to bleomycin, as well as an increased recruitment of monocytes/macrophages to the lungs. Importantly, the lack of Fbxw7 increased the secretion of TGF-β1 through ubiquitin-dependent degradation [116]. This study highlights, therefore, the protective role of Fbxw7 in lung fibrosis through the inhibition of macrophage profibrotic responses.

miRNAs (microRNAs) are short, non-coding RNAs known for their role in post-transcriptional regulation of gene expression via the degradation of target RNA. The involvement of miRNAs in IPF pathogenesis, notably in the transcriptional regulation of epithelial cells and fibroblasts, has been revealed. Drosha ribonuclease III (DROSHA) is a ribonuclease enzyme that participates in the nuclear biogenesis of miRNAs. Cho et al. have elucidated the role of DROSHA in macrophage activation in IPF. They first reported an upregulated expression of DROSHA in AMs from IPF patient lungs in comparison with control donors. Moreover, DROSHA protein significantly colocalizes with absent in melanoma 2 (AIM2) inflammasome in AMs. Similar results were observed in the bleomycin mouse model, suggesting a potential link between DROSHA induction and AIM2 activation. As a matter of fact, DROSHA deletion in mouse macrophages confirmed the role of DROSHA in promoting AIM2 inflammasome activation through a miRNA-mediated mechanism [117]. The involvement of exosomal miRNAs was also studied by Guiot et al. in IPF. Exosome isolation from sputum and plasma revealed higher expression of miR-142-3p in sputum-derived and plasma-derived exosomes from IPF patients than in healthy donors. Correlation analysis identified macrophages as the primary source of exosomes in IPF. Interestingly, macrophage-derived exosomes can transfer miR-142-3p to fibroblasts and epithelial cells and consequently reduce the profibrotic responses of these target cells [118]. Taken together, macrophage capacity to release miR-142-3p-loaded exosomes represents a potential antifibrotic mechanism that allows the downregulation of IPF.

The Forkhead box M1 (FOXM1) is a proliferation-associated transcription factor. In IPF, FOXM1 is abnormally expressed in fibroblasts and seems to be involved in epithelial-to-mesenchymal transition [119,120]. Goda et al. recently reported that FOXM1 is inducible on AMs from IPF patients within fibrotic scars, while the expression is not detectable in healthy donor macrophages. In mice, myeloid-specific deficiency of FOXM1 was associated with an increased accumulation of collagen in bleomycin-treated mice as well as an increased number of αSMA^+^ myofibroblasts. Furthermore, the lack of FOXM1 potentiates the expression of profibrotic cytokines in AMs, leading to fibroblast activation. Mechanistically, the study shows that *Dusp1* transcriptional regulation by FOXM1 inhibits p38 MAPK phosphorylation in macrophages [121]. This could explain, therefore, the anti-fibrotic role of FOXM1 in macrophages during pulmonary fibrosis. It is worthwhile mentioning here that previous studies revealed that FOXM1 inhibition in fibroblasts and epithelial cells has a protective effect on lung fibrosis. This suggests that FOXM1 could have opposite roles in different cell types [119,120].

Fra-2 is known to be highly implicated in cellular transcriptional regulation and was described in several lung pathologies [122]. In particular, Fra-2 transgenic mice develop spontaneous lung fibrosis with high levels of IL-4 [123]. The role of this transcription factor was elucidated in IPF by Ucero et al. They showed that Fra-2 transgenic mice have an increased expression of M2 macrophage markers, suggesting an enrichment in profibrotic macrophages. Using different preclinical models, it was shown that macrophages that express Fra-2 are highly involved and required for the development of pulmonary fibrosis. The study also reported the production of collagen VI by profibrotic macrophages in a Fra-2-dependent manner. These data were confirmed in lung biopsies from IPF patients that display elevated levels and colocalization of Fra-2 and collagen VI in both alveolar and interstitial macrophages when compared with healthy tissues [78]. Altogether, the study suggests that Fra-2 strengthens the profibrotic response of macrophages in lung fibrosis.

Lee et al. identified a new genetic modifier of TGF-β1 pathway that is involved in macrophage activation during pulmonary fibrosis. A transgenic mouse with inducible expression of bioactive TGF-β1 was generated and bred onto different strains. Based on haplotype-based computational genetic mapping and mRNA profiling, Laminin α1 (Lama1) was chosen for analysis among other candidates due to its high genetic effect on TGF-β1-induced pulmonary fibrosis. Lama1 aggravates pulmonary fibrosis, as revealed by collagen deposition and collagen-related gene expression. Moreover, Lama1 is implicated in the M2 polarization of macrophages and enhances their fibrotic activity. Unexpectedly, however, Lama1 is expressed in pulmonary macrophages from C57 TGF-β1 transgenic mice but not in BALB/c mice, revealing, therefore, a strain-specific expression in mice. Human data confirmed that Lama1 expression is upregulated in fibrotic lungs of IPF patients in comparison with healthy donors and that macrophages and fibroblasts are the major Lama1^+^ cells in the lungs [124].

It is becoming evident that transcriptional and epigenetic machinery is crucial to program macrophage differentiation, polarization, and activation. The dysregulation of gene expression critically participates in the development of IPF, and a better understanding of the underlying mechanisms may lead to significant progress. The specific mechanisms and therapeutic possibilities, however, still require additional study.

### 3.4. Macrophage Subsets and Cytokines/Chemokines

Circulating monocytes are drawn towards the wounded area of the lung following injury and differentiate into either the pro-inflammatory/cytotoxic (M1) or the anti-inflammatory/wound-healing (M2) macrophage subpopulations. Apart from the classical functional subsets, new studies are identifying novel subsets of lung macrophages according to the expression of distinct surface markers in order to uncover their role in the development of IPF. In particular, most research focuses on the secretory profile of these different subsets, offering, therefore, relevant insights into IPF etiology. In this section, we will highlight recent studies that investigated novel macrophage subsets in IPF as well as macrophage-derived cytokines/chemokines.

#### 3.4.1. Subsets

Integrin α M (CD11b) is an adhesion molecule involved in various cell functions and migrations. Once lung injury occurs, CD11b^+^ monocytes/macrophages migrate into the injured lung interstitial compartments, polarize into CD206^+^ M2 macrophages, and promote pulmonary fibrosis. For the purpose of investigating the precise role of CD11b^+^ monocytes/macrophages, Wan et al. conditionally depleted CD11b cells in a CD11b-diphtheria toxin receptor (CD11b-DTR) mouse. CD11b depletion eventually inhibited the polarization of macrophages in the fibrotic lungs. Sphingosine-1-phosphate (S1P) and its receptor S1pr2 have been implicated in pulmonary fibrosis, and they demonstrated that CD11b deficiency inhibits sphingosine 1-phosphate receptor 2 (S1PR2)/sphingosine kinase 2 (SphK2) signaling during pulmonary fibrosis. Additionally, SphKs axis was found to be necessary for TGF-β1 induced myoblast-to-myofibroblast trans-differentiation [125]. In the same context, McCubbrey et al. focused on macrophages that display high expression of CD11b. Interestingly, CD11b^hi^ macrophages were found to be increased in the lungs and to be distinct from SiglecF^hi^ macrophages as they show higher expression of pro-fibrotic chemokines, including CCL2, CCL12, and CCL24. The latter promotes the survival of fibroblasts, while CCL2 stimulates the proliferation and production of collagen. CD11b^hi^ macrophages were found to have a higher expression of epithelial and fibroblast proliferative factors. Therefore, a novel transgenic hCD68rtTA system was created to selectively target CD11b^hi^ macrophages without affecting SiglecF^hi^ macrophages. The survival of CD11b^hi^ macrophages was manipulated by deleting the anti-apoptotic protein, cellular FADD-like IL-1b–converting enzyme–inhibitory protein (c-FLIP) upon administration of doxycycline. Hence, the loss of CD11b^hi^ macrophages protected mice from the development of lung fibrosis. Further, hCD68rtTA targets both alveolar and tissue CD11b^hi^ macrophages, whereas alveolar and tissue CD11b^hi^ macrophages are different subsets, and it will be interesting to investigate their pro-fibrotic effects separately [126].

Morse et al. investigated macrophage subsets in human fresh lung explants from various fibrotic lobes that resembled the disease. The upper fibrotic lobes showed early disease, whereas the lower fibrotic lobes reflected late disease conditions. The various degrees of fibrosis and disease severity were indicated by a graded pattern of cell type changes between the normal, upper, and lower lobes of IPF. They found three discrete macrophage subsets: one expressing monocyte markers, one highly expressing FABP4 and INHBA (FABP4^hi^), and one expressing SPP1 and MERTK (SPP1^hi^). Furthermore, Morse et al. analyzed the Secreted Phosphoprotein 1 (SPP1) marker on macrophages from healthy and IPF lungs and discovered that SPP1^hi^ macrophages in IPF originate from a pre-existing macrophage population. *SPP1* expression, on the other hand, rises considerably in macrophages found in fibrotic IPF lower lobes. At the same time, *Spp1* deletion in bleomycin mice downregulated the expression of collagen type 1 and MMP2, ultimately ameliorating fibrosis. SPP1^hi^ macrophages were suggested to constitute a pro-fibrotic macrophage population in IPF lungs. SPP1^hi^ macrophages in fibrotic lower lobes exhibited substantially elevated *SPP1* and *MERTK* expression. FABP4 expression in macrophages was associated with pro-inflammatory macrophages and the release of IL-1β. Alternatively, in IPF lungs, FABP4^hi^ macrophages account for the majority of macrophages in alveoli and may differentiate into SPP1^hi^ macrophages. FCN1^hi^ macrophages form the third group found largely in the interstitial compartment, expressing markers closely related to monocytes. The study suggested that SPP1^hi^ macrophages contribute to lung fibrosis in IPF, and therapeutic strategies targeting MERTK and macrophage proliferation might provide novel treatment options for the disease [58].

Nouno et al. worked on lung tissue biopsies from patients with IPF and autopsies from AE IPF to analyze the association between pulmonary accumulation of M2-like macrophages and survival in IPF patients. Subset identification was based on three markers: CD68 (pan-macrophage marker), CD163, and CD204 (M2-like macrophage markers). They found that lung tissues from IPF patients contained more CD68^+^, CD163^+^, and CD204^+^ cells and had higher CD163^+^/CD68^+^ and CD204^+^/CD68^+^ cell ratios than those from the control group. In situ hybridization of TGF-β1 mRNA was performed and revealed a histological co-localization with stained CD163^+^ cells in lung tissue sections of IPF patients. Poorer clinical outcomes in IPF patients were associated with the elevation of CD163^+^ and CD204^+^ macrophages. The study concluded that suppressing macrophage activation or macrophage-derived TGF-β1 production might be a therapeutic target against IPF [127].

Li et al. observed that bleomycin-treated mice had more S100A4^+^ macrophages in their lung tissues. During the inflammatory phase, S100A4^+^ macrophages were found to be the most prominent cells stimulating fibroblast activation. Whereas S100A4 deficiency (S100A4^−/−^) or blocking of S100A4 using a neutralizing antibody has reduced fibrosis. They further performed the adoptive transfer of S100A4^+^ and S100A4^−^ macrophages in S100A^−/−^ mice and found that the transfer of S100A4^+^ macrophages causes the development of fibrosis. Additionally, S100A4 protein levels and the number of S100A4^+^ macrophages were found to be correlated with the occurrence of IPF in patients [128].

#### 3.4.2. Cytokines/Chemokines

Besides classical macrophage-derived cytokines, recent studies demonstrated the capacity of lung macrophages to modulate the progression of IPF via a wide spectrum of cytokines, including IL-9, IL-11, and IL-37, and chemokines such as CCL17 and CXCL13.

Sugimoto et al. explored the effect of IL-9 on pulmonary fibrosis using the silica-induced mouse lung fibrosis model. They discovered elevated levels of IL-9 in BAL of a silica-induced fibrotic group, while neutralization of IL-9 with an anti-IL9 neutralization antibody suppressed the inflammation and fibrosis. Similarly, in human studies, cells in IPF lungs had significant levels of IL-9 expression compared to healthy controls. Immunohistochemistry analysis clearly showed that mainly CD4^+^ and AMs express IL-9. The study also showed that IL-9 induces pro-fibrotic TGF-β production by AMs, which has been known to be implicated in pulmonary fibrosis exacerbation [129]. 

Kortekaas et al. investigated the function of IL-11 in the pathogenesis of IPF. The ability of IL-11 to initiate the differentiation of fibroblasts into myofibroblasts and to induce the production of collagen has been associated with the development of fibrosis. It’s interesting to note that in this study, the IL-11 protein was also found to be expressed by AMs. Although the precise function of IL-11 in AMs and its connection to IPF are still unknown, more research will be intriguing [130]. 

Kim et al. demonstrated the effect of the anti-inflammatory cytokine IL-37 in pulmonary fibrosis. They found that IL-37 protein was expressed in AECs and AMs in healthy controls but significantly reduced in patients with IPF. As lung cell apoptosis is very important in lung fibrosis, they performed siRNA-mediated silencing of IL-37 in primary mouse AECs or A549 cells and found that IL-37 was protective against oxidative stress–induced AEC death. Further, IL-37 was found to reduce the constitutive expression of fibronectin and collagen in IPF lung fibroblasts. IL-37 inhibits fibroblast proliferation and downregulates TGF-β1 signaling. Autophagy is an intrinsic cellular defense mechanism in innate and adaptive immune systems. Kim et al. checked how IL-37 regulates autophagy, especially in lung fibrosis, and found that IL-37 suppresses mTOR, the mediator of PI3K/AKT signaling that inhibits macroautophagy. Further research revealed that the anti-fibrotic properties of IL-37 were reversed when autophagy was inhibited by 3-MA (3-methyladenine). The study concluded that, in addition to autophagy, the blockage of TGF-β1 signaling and the inhibition of PI3K/AKT signaling contributed to the anti-fibrotic effect of IL-37 [131].

CCL17 has been associated with various inflammatory disorders such as dermatitis, allergic asthma, atherosclerosis, colitis, and arthritis. CCL17 has recently been revealed to have an important role in the etiology of fibrotic disorders. Wang et al. demonstrated that the levels of CCL17 were increased in the lungs of IPF patients and mice with bleomycin-induced IPF. AMs were identified as the primary source of elevated levels of CCL17 in the lungs. Furthermore, silencing CCR4 expression significantly reduced the expression of α-SMA and COL1 in CCL17-treated fibroblasts, suggesting that CCR4 on the fibroblast membrane was responsible for CCL17-mediated fibroblast activation. Moreover, the knockdown of CCR4 by CCR4-siRNA or blockade by CCR4 antagonist C-021 was able to ameliorate pulmonary fibrosis pathology in mice. The study showed that CCL17 is a pro-fibrotic mediator of lung fibroblasts, suggesting that CCL17 or CCR4 inhibition may serve as an effective strategy to suppress lung fibroblast activation and attenuate pulmonary fibrosis progression [132].

Bellamri et al. investigated CXCL13 in biopsies from IPF patients. Multiplex immune-fluorescence staining showed that CXCL13 was present in CD68 and CD206-positive AMs. In vitro studies revealed that both canonical and non-canonical NF-kB signaling increased the production of CXCL13 in LPS-stimulated and monocyte-derived macrophages. LPS rapidly induces TNF-α and IL-10 expression, which then triggers the NF-kB and JAK/STAT pathways, respectively, to facilitate *CXCL13* gene activation. They concluded that TNF-α and IL-10 are mediators of LPS-induced *CXCL13* gene expression in AMs [36].

Apart from classical macrophages, various other macrophage subsets have been associated with distinct phenotypes, functions, and secretomes. As a matter of fact, targeting cytokine-specific pathways or depleting specific subsets seems to be an effective way to attenuate fibrosis. Thus, further clinical and animal studies warrant novel approaches to modulating mediators and fibrotic macrophage subsets. 

## 4. Conclusions

Over the last decade, our insights into immune cell implications in the pathogenesis of lung fibrosis have been profoundly revolutionized by the use of new technologies such as scRNA-seq and proteomic imaging. This review focuses on recently discovered molecular mechanisms and pathological changes associated with pulmonary macrophages in lung fibrosis. A pivotal role for macrophages in promoting the progression of lung fibrosis has been confirmed in IPF patients as well as in mouse models. Targeting macrophages in IPF is now the subject of clinical research and trials. However, different factors complicate the task since macrophages do not consist of one homogeneous cell population with a unique phenotype and functional properties. Moreover, macrophages are highly plastic cells that undergo functional polarization based on complex pathways and regulatory networks. Therefore, strategies based on the reprogramming of pro-fibrotic macrophages toward an anti-fibrotic phenotype should be highly specific in targeting relevant subsets without compromising others. In this context, some immune receptors can be targeted to modulate macrophage fibrotic responses. The suggested alignment of lung fibrosis diseases with cancer further supports the potential effectiveness of these therapeutic options in IPF. Other studies build upon the impact of metabolic, transcriptional, and epigenetic factors in shaping macrophage activity during the development of fibrosis. Although very diverse, all perspectives will drive us toward a better understanding of macrophage-related mechanisms in IPF and might provide the potential for paradigm shifts in treatment.

## Figures and Tables

**Table 1:** Role of main innate and adaptive immune cells in IPF.

Innate Immune Cells	Role in IPF	References
Neutrophils	Neutrophil elastase (NE) promotes fibrosis and tissue remodeling. Forced vital capacity values and bronchoalveolar lavage (BAL) neutrophil counts have an inverse relationship. Neutrophil extracellular traps (NETs) are found to be associated with fibrosis.	[15,16,17,18,19,20]
Macrophages	Pro-inflammatory and anti-fibrotic properties associated with M1 macrophages. Anti-inflammatory, pro-fibrotic, and tissue-regenerating properties are associated with M2 macrophages.	[14,21,22,23,24]
Monocytes	Progenitor cells for pro-fibrotic macrophages and fibrocytes. Release (pro-fibrotic) inflammatory cytokines. Increased monocyte count is correlated with poorer survival.	[10,25,26,27]
Fibrocytes	Fibrocytes contribute to fibroblast-mediated tissue remodeling.	[27,28]
Myeloid-derived suppressor cells (MDSCs)	Increased MDSC numbers are associated with poor lung functions, severe pulmonary hypertension, and increased regulatory T cells. Involved in pro-fibrotic and immune-dysregulated environments.	[26,27,29]
Type-2 innate lymphoid cells (ILC2s)	Increased ILC2 count in IPF patients’ BAL associated with an enhanced type-2 immune environment. ILC2s potentiate ECM synthesis and tissue remodeling via IL-13 production.	[27,30,31,32]
Dendritic cells (DCs)	Immature DCs accumulate in regions of epithelial hyperplasia and fibrotic lesions. Mature DCs are concentrated in lymphoid follicles along with T and B cells in IPF patients. Possibly involved in ongoing inflammation in IPF lungs.	[10,33,34]
**Adaptive Immune Cells**		
B cells	Increased number of IgA^+^ memory B cells and plasmablasts in the blood and lungs of IPF patients. High levels of B cell activation factor (BAFF) and CXCL13 in the serum of IPF patients. CpG and β-glucan stimulation of B cells promotes inflammatory and fibrotic changes in IPF patients.	[35,36,37,38]
Th1 cells	Attenuate fibrosis via the production of IFN-γ.	[26,38,39]
Th2 cells	Dominant in IPF and antagonizes Th1 cells. Enhance fibrosis through the production of type 2 cytokines such as IL-4, IL-5, and IL-13. IL-4 and IL-13 stimulate (myo) fibroblast activation and proliferation while predisposing macrophages to a pro-fibrotic phenotype.	[26,38,39,40]
Th9 cells	Unclear role in IPF etiology contradictory effects of Th9 cells and IL-9 have been observed in the development of fibrosis.	[10,41,42]
Th17 cells	Pro-fibrotic function via the production of IL-17, which stimulates fibroblast proliferation and collagen secretion.	[17,38,39,41,43]
Regulatory T cells (Tregs)	Tregs have opposing roles in the progression of IPF. Promotion or inhibition depends on the disease stage.	[38,44,45,46]

## Data Availability

Not applicable.
